# Dynamics of immune responses following duck Tembusu virus infection in adult laying ducks reveal the effect of age-related immune variation on disease severity

**DOI:** 10.1016/j.psj.2024.104731

**Published:** 2024-12-25

**Authors:** Teerawut Nedumpun, Kanana Rungprasert, Patchareeporn Ninvilai, Benchaphorn Limcharoen, Wikanda Tunterak, Duangduean Prakairungnamthip, Navapon Techakriengkrai, Wijit Banlunara, Sanipa Suradhat, Aunyaratana Thontiravong

**Affiliations:** a Department of Veterinary Microbiology, Faculty of Veterinary Science, Chulalongkorn University, Bangkok, Thailand, 10330; b Center of Excellence for Emerging and Re-emerging Infectious Diseases in Animals (CUEIDAs), Faculty of Veterinary Science, Chulalongkorn University, Bangkok, Thailand, 10330; c Avian Veterinary Services, CPF (Thailand) Public Company Limited, Bangkok, Thailand; d Department of Anatomy, Faculty of Veterinary Science, Chulalongkorn University, Bangkok, Thailand, 10330; e Department of Pathology, Faculty of Veterinary Science, Chulalongkorn University, Bangkok, Thailand, 10330; f Center of Excellence in Animal Vector-Borne Diseases, Veterinary Parasitology Unit, Department of Veterinary Pathology, Faculty of Veterinary Science, Chulalongkorn University, Bangkok, Thailand, 10330; g Center of Excellence of Systems Microbiology, Faculty of Medicine, Chulalongkorn University, Bangkok, Thailand, 10330

**Keywords:** Innate immune response, adaptive immune response, age, duck, duck Tembusu virus

## Abstract

Duck Tembusu virus (DTMUV), an emerging avian pathogenic flavivirus, is notably associated with neurological disorders and acute egg drop syndrome in ducks. We previously demonstrated that the susceptibility of ducks to DTMUV infection varies significantly with age, with younger ducks (4-week-old) exhibiting more severe disease than older ducks (27-week-old). However, the immunological mechanisms underlying these age-related differences in disease severity remain unclear. In this study, we investigated the dynamics of immune responses following DTMUV infection in adult laying ducks (27-week-old) and compared them to our previous findings on young ducks (4 weeks old). The numbers of T helper, cytotoxic T, B, and non-T and B lymphocytes, as well as neutralizing antibody levels, were measured in parallel with DTMUV loads in the blood and target organs. Our results revealed that the number of non-T and B lymphocytes/myeloid cells in 27-week-old adult laying ducks infected with DTMUV remained consistently stable throughout the observation period, in contrast to findings in 4-week-old younger ducks, where myeloid cell responses were implicated in disease progression. Regarding lymphocyte responses, unlike in 4-week-old younger ducks, only cytotoxic T lymphocyte responses in 27-week-old older ducks showed a significant negative correlation with the reduction of viremia and viral loads in target organs, indicating their role in controlling viral replication in older ducks. Additionally, 27-week-old adult laying ducks infected with DTMUV exhibited high levels of neutralizing antibodies, which were significantly correlated with reduced viral loads in blood and target organs. Overall, the presence of robust DTMUV-specific neutralizing antibody and CTL responses, along with a finely tuned myeloid cell response likely plays a significant role in controlling severe neurological outcomes in 27-week-old adult laying ducks. This study highlights the age-related differences in immune responses following DTMUV infection, which potentially contribute to the varying disease severity among ducks of different ages. Understanding the interplay between the host and DTMUV provides significant implications for disease management strategies and vaccine development.

## Introduction

Duck Tembusu virus (DTMUV) is an emerging avian pathogenic flavivirus that primarily causes severe neurological disorders in young ducks, and acute egg drop syndrome in adult laying ducks, leading to significant economic losses in the duck producing industry (Hamel et al., 2021). This disease can manifest with a wide range of severities, presenting mild to severe clinical symptoms, mostly depending on the viral genotype and duck age (Feng et al., 2020; Ninvilai et al., 2020; Tunterak et al., 2023). Recent studies have revealed marked differences in pathogenicity and disease severity among different genotypes of DTMUV (Feng et al., 2020; Zhu et al., 2022; Tunterak et al., 2023). In addition to virus-related factors, it is widely recognized that duck age is another key factor involved in the virulence and pathogenicity of DTMUV (Sun et al., 2014; Li et al., 2015; Ninvilai et al., 2020). However, the underlying mechanism for this age-related factor remains largely unknown.

Several previous studies have consistently shown differences in pathogenicity and disease severity of DTMUV infection between ducks of different ages, indicating age-related susceptibility to DTMUV in ducks (Sun et al., 2014; Li et al., 2015). Correspondingly, we also demonstrated that DTMUV induced more severe disease and higher mortality in younger ducks (1 and 4 weeks old) than older ducks (27 weeks old) (Ninvilai et al., 2020). The variation in the outcome of DTMUV infection in ducks of different ages may be attributed to immune-mediated mechanisms. However, the immunological mechanisms contributing to the differences in disease severity between young and adult ducks remains unclear. Presently, studies on the immune responses against DTMUV in ducks have primarily focused on young ducks (Li et al., 2015; Thontiravong et al., 2022). Limited information is available on the immune responses to DTMUV infection, especially adaptive cellular immune response, in adult laying ducks. To further investigate the effects of age on immune responses to DTMUV infection, we evaluated the dynamics of T helper lymphocyte (Th, CD3^+^CD4^+^), cytotoxic T lymphocyte (CTL, CD3^+^CD8^+^), B lymphocyte (CD3^-^IgY^+^), and non-T and B (CD3^-^ IgY^-^) lymphocyte responses, as well as neutralizing antibody responses, following DTMUV infection in adult laying ducks (27 weeks old). The results were then compared to our previous report on young ducks (4 weeks old) (Thontiravong et al., 2022).

## Materials and methods

### Virus

DTMUV strain DK/TH/CU-1 (GenBank accession number KR061333), a member of the genus *Flavivirus* within the family *Flaviviridae*, was isolated from DTMUV-infected ducks in Thailand and used in this study (Thontiravong et al., 2015). This virus belongs to the DTMUV cluster 2, the predominant cluster circulating in duck populations in Asia (Ninvilai et al., 2019). The virus was propagated and titrated in 9-day-old embryonated duck eggs, as previously described (Ninvilai et al., 2020). The virus was stored at -80°C until use.

### Animal study

To evaluate the dynamics of immune responses following DTMUV infection in adult laying ducks, we utilized whole blood and sera from DTMUV-infected and non-infected control adult laying ducks (27 weeks old), obtained from our previously described pathogenesis study (Ninvilai et al., 2020). Briefly, 35 specific pathogen-free (**SPF**) ducks at 27 weeks of age were inoculated intranasally and intramuscularly with 10^5^ 50% embryo lethal dose (**ELD_50_**)/ml of DTMUV (strain DK/TH/CU-1). An additional group of 35 ducks was mock inoculated with allantoic fluid from SPF duck eggs using the same procedure, serving as the non-infected control group. These ducks were obtained from a private research farm operating under high biosecurity standards and were confirmed to be free from common duck viruses, including DTMUV, through virus-specific RT-PCR/PCR and serological assays. Heparinized whole blood from five ducks in each group were collected at 0, 3, 5, 7, 9, 14, and 21 days post-inoculation (**dpi**) to study the cellular immune response, while sera were collected for serum neutralization (**SN**) testing at 1, 3, 5, 7, 9, 14, and 21 dpi. Additionally, blood samples and target organs, including brains and spleens, were collected for viral load determinations at 1, 3, 5, 7, 9, 14, and 21 dpi. DTMUV loads in blood and target organs were measured by DTMUV E-specific qRT-PCR, in parallel with immunological analyses, and this data was retrieved from our previous DTMUV pathogenesis study (Ninvilai et al., 2020). The animal experiment was performed in the ABSL-2 containment facility at Chulalongkorn University Laboratory Animal Center (**CULAC**) and was conducted under the approval of Chulalongkorn University Animal Care and Use Committee (approval number 1673012). The immune responses to DTMUV in 27-week-old adult laying ducks were then compared to our previous report on 4-week-old young ducks, which was conducted in the same manner as described in this study (Thontiravong et al., 2022).

### Isolation of duck peripheral blood mononuclear cells

Peripheral blood mononuclear cells (**PBMC**) were isolated from heparinized whole blood obtained from both DTMUV-infected and non-infected 27-week-old ducks. This process was carried out by density gradient centrifugation for 20 minutes at 1,000xg using Isoprep separation medium (Robbins Scientific Co., CA, USA) following the manufacturer's instructions. Subsequently, the cells were resuspended in complete RPMI medium and subjected to immunofluorescent staining.

### Immunofluorescent staining and flow cytometric analysis

Frequencies of CD4^+^ and CD8^+^ T, B, and non-T and B lymphocyte subpopulations in the PBMC of both DTMUV-infected and non-infected 27-week-old ducks were evaluated by flow cytometric analyses, as described previously (Kothlow et al., 2005; Thontiravong et al., 2022). Briefly, PBMC were seeded in a 96-well round-bottom plate at a concentration of 1 × 10^6^ cells/well in the complete RPMI medium, then washed twice with FACS buffer (PBSA supplemented with 1% FBS and 0.1% sodium azide). The cells were subsequently stained with 3 combination of antibodies to identify duck CD4^+^ lymphocytes (rat anti-human CD3ε conjugated with Alexa Fluor 647; clone CD3-12, isotype rat IgG1 and mouse anti-duck CD4; clone Du CD4-2, isotype mouse IgG2a), duck CD8^+^ lymphocytes (rat anti-human CD3ε conjugated with Alexa Fluor 647 and anti-duck CD8α; clone Du CD8-1, isotype mouse IgG2b), and duck B lymphocytes (mouse anti-duck IgY light chain; clone 14A3, isotype mouse IgG1 and rat anti-human CD3ε conjugated with Alexa Fluor 647) for 30 min at 4°C in the dark. All antibodies were purchased from Bio-Rad, CA, USA. After washing with FACS buffer, isotype specific secondary antibodies, including goat anti-mouse IgG2a FITC, goat anti-mouse IgG2b PE-Cy7, and goat anti-mouse IgG1 PE (Southern Biotech, AL, USA), were added and incubated in the dark at 4°C for 30 minutes. Cells stained with isotype control antibodies were included as the background cut-off. The fluorescence minus one (FMO) staining controls were also performed during assay validation. The cells were gated at a minimum of 5 × 10^4^ cell/events for each analysis, and samples were processed through an FC 500 MPL (Beckman Coulter, CA, USA), with subsequent analysis using FlowJo software (Tree Star Inc., OR, USA). The cells were gated into CD3^+^CD4^+^ (Th, T helper lymphocyte), CD3^+^CD8^+^ (CTL, cytotoxic T lymphocyte), CD3^-^IgY^+^ (B lymphocyte), and CD3^-^IgY^-^ (non-T and B lymphocyte) subpopulations. The absolute numbers of each subpopulation in the PBMC, reflecting actual changes induced by DTMUV infection, were calculated using percentages obtained from flow cytometry combined with the total PBMC count, as previously described (Solomos et al., 2016; Thontiravong et al., 2022).

### Serum neutralization test

The level of DTMUV-specific neutralizing antibodies in serum samples from 27-week-old ducks was determined by serum neutralization (SN) test using the BHK-21 cells and DK/TH/CU-1, as previously described (Tunterak et al., 2018). The SN test is a common serological method used to detect the presence and magnitude of neutralizing antibodies, which are capable of preventing viral infectivity (Hedman and Nurmi, 2021). Briefly, triplicate serial two-fold dilutions of heat inactivated sera were incubated with 100 TCID_50_ of DK/TH/CU-1 for 1 h at 37°C. Following incubation, the virus-serum mixture was added to a 96-well plate containing BHK-21 cells. The cells were then incubated at 37°C and were monitored for the development of cytopathic effects (CPE) daily for 5 days. Reference DTMUV antibody positive and negative sera, uninfected BHK-21 cells, and back titration of used virus served as controls. SN antibody titers were expressed as the reciprocal of the highest serum dilution capable of inhibiting CPE.

### Statistical analysis

Statistical analysis was conducted to assess the survival probability in relation to the occurrence of neurological signs and the incidence of mortality following DTMUV infection. The immunological data were tested for normality distribution using the Shapiro-Wilk test. Data were analyzed using a two tailed, unpaired Student's *t-*test. Correlations were evaluated by Pearson's correlation analysis. All statistical analyses were performed using the GraphPad Prism 6.0 software (GraphPad Software Inc. La Jolla, CA). All P < 0.05 were considered statistically significant.

## Results

### Mild clinical disease in 27-week-old adult laying ducks following duck Tembusu virus inoculation

**Fig. 1:**
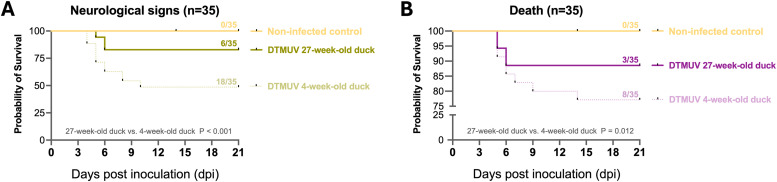
Mild duck Tembusu virus (DTMUV)-associated clinical disease observed in 27-week-old adult laying ducks. Statistical analysis was conducted to assess the survival probability in relation to the occurrence of neurological signs (A) and the incidence of mortality (B) following DTMUV infection in 27-week-old and 4-week-old ducks. All the data were retrieved from a previous DTMUV pathogenesis study (Ninvilai et al., 2020) and re-analyzed to evaluate survival probability. Significant differences between groups are indicated as P < 0.001 and P = 0.012.

As described in our previous study (Ninvilai et al., 2020), 27-week-old adult laying ducks showed mild neurological signs, while 4-week-old young ducks displayed severe neurological signs, including ataxia, reluctance to walk, and paralysis. The neurological signs in DTMUV-inoculated adult laying ducks were only observed at 5-6 dpi, and all ducks recovered by 9 dpi (Fig. 1A). In DTMUV-inoculated 4-week-old young ducks, the neurological signs were markedly observed from 4 to 10 dpi, with some recovering by 12 dpi (Fig. 1A). After DTMUV infection, the cumulative incidence of neurological signs in affected younger ducks (18/35) was significantly higher than that observed in older ducks (6/35) (P < 0.001) (Fig. 1A). Only 3 out of 35 inoculated 27-week-old adult laying ducks died at 5-6 dpi, whereas a significantly higher mortality rate was observed among inoculated 4-week-old young ducks (P = 0.012), with 8 out of 35 dying between 5 and 12 dpi (Fig. 1B). Additionally, our previous study revealed that the severity of pathological changes in immune organs of 4-week-old young ducks, including splenomegaly, bursal atrophy, and thymic hemorrhage, was higher than in 27-week-old adult laying ducks, where only mild splenomegaly was observed (Ninvilai et al., 2020). Taken together, the severity of DTMUV-induced disease was notably higher in younger ducks (4-week-old) than in older ducks (27-week-old), as evidenced by increased morbidity and mortality rates.

### Dynamics of lymphocyte responses and their correlations with DTMUV loads in 27-week-old adult laying ducks

**Fig. 2:**
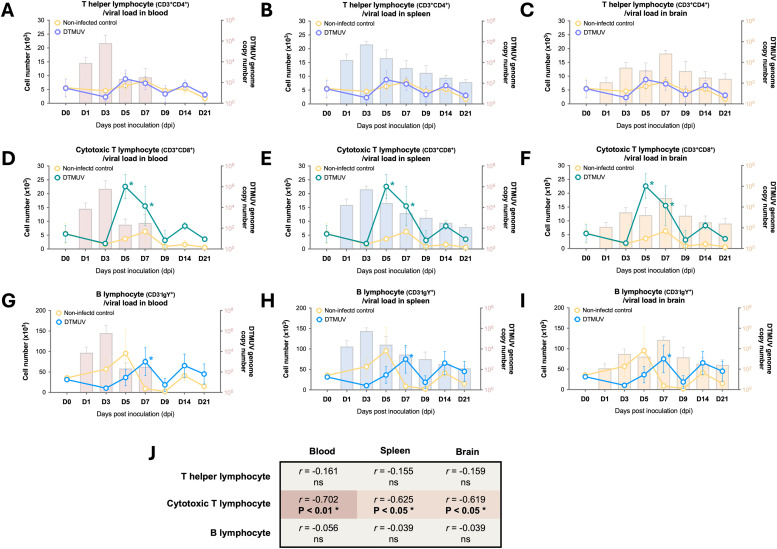
Dynamic profiles of lymphocyte responses in 27-week-old adult laying ducks infected with duck Tembusu virus (DTMUV). (A-I) Kinetics of T helper lymphocyte (Th, CD3^+^CD4^+^), cytotoxic T lymphocyte (CTL, CD3^+^CD8^+^), and B lymphocyte (CD3^-^IgY^+^) numbers in the peripheral blood of DTMUV-infected and non-infected control ducks at the indicated time points. The relationships between viral loads in blood (A, D, G) and target organs (B-C, E-F, H-I), alongside lymphocyte responses, are shown throughout the course of DTMUV infection. DTMUV genome copy numbers in blood and target organs are displayed as bars, while the absolute numbers of lymphocyte subpopulations in the PBMC of DTMUV-infected and non-infected control ducks are shown as lines. All viral load data were retrieved from a previous DTMUV pathogenesis study (Ninvilai et al., 2020). Asterisks (*) indicate significant differences (P < 0.05) compared to the non-infected controls. (J) Correlations of lymphocyte numbers with viral loads in blood and target organs. "ns" indicates not significant, and asterisks (*) indicate significant differences (P < 0.05 and P < 0.01).

To investigate the dynamics of lymphocyte responses following DTMUV infection in 27-week-old adult laying ducks, the absolute numbers of T helper lymphocyte (Th, CD3^+^CD4^+^), cytotoxic T lymphocyte (CTL, CD3^+^CD8^+^), and B lymphocyte (CD3^-^IgY^+^) subpopulations in the PBMC were analyzed using flow cytometry. The gating strategy of duck lymphocyte subpopulations had been described in our previous report (Thontiravong et al., 2022). The results demonstrated that there was no significant difference in the numbers of Th lymphocytes between the inoculated ducks and the non-infected control ducks (Fig. 2A-C). The numbers of B lymphocytes in inoculated ducks paralleled those of Th lymphocytes, displaying levels comparable to the non-infected control group (Fig. 2G-I). Interestingly, these findings contrasted with those from our previous report on 4-week-old young ducks, where the numbers of Th and B lymphocytes in the PBMC of infected younger ducks increased significantly from 5 to 9 dpi compared to the non-infected controls (Thontiravong et al., 2022). For CTL responses, the numbers of cells in DTMUV-inoculated 27-week-old adult laying ducks increased significantly (P < 0.05) between 5 and 7 dpi, and then returned to levels similar to those of the non-infected ducks by 9 dpi (Fig. 2D-F). This trend was relatively similar to that observed in 4-week-old young ducks (Thontiravong et al., 2022).

To evaluate the role of lymphocyte responses on DMTUV control, the correlations between cellular numbers of each lymphocyte subpopulation and DTMUV loads in blood and target tissues (spleen and brain) were analyzed. Interestingly, a significantly negative correlation was only observed between CTL numbers and viral loads in blood (*r* = -0.702, P < 0.01), spleen (*r* = -0.625, P < 0.05), and brain (*r* = -0.619, P < 0.05) from 5 to 21 dpi (Fig. 2J). This finding contrasted with those observed in 4-week-old young ducks where a negative association was observed between CTL numbers and viral loads in the blood and spleen, but not in the brain (Thontiravong et al., 2022). Altogether, these observations suggested that CTL responses potentially play a critical role in controlling DTMUV loads in 27-week-old adult laying ducks, thereby reducing clinical outcomes after DTMUV infection.

### Dynamics of non-T and B lymphocyte responses and their correlations with DTMUV loads in 27-week-old adult laying ducks

**Fig. 3:**
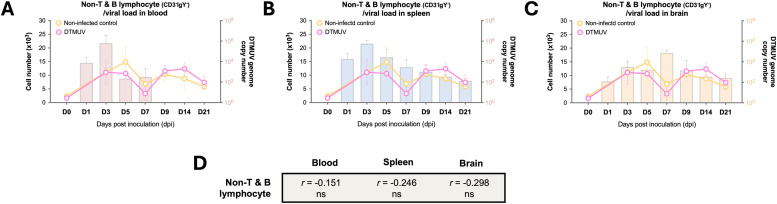
Dynamic profiles of non-T and B lymphocyte responses in 27-week-old adult laying ducks infected with duck Tembusu virus (DTMUV). (A-C) Kinetics of non-T and B lymphocyte/myeloid cell (CD3^-^IgY^-^) numbers in the peripheral blood of DTMUV-infected and non-infected control ducks at the indicated time points. The relationships between viral loads in blood (A) and target organs (B-C), alongside non-T and B lymphocyte/myeloid cell responses, are shown throughout the course of DTMUV infection. DTMUV genome copy numbers in blood and target organs are displayed as bars, while the absolute numbers of non-T and B lymphocyte/myeloid cell subpopulations in the PBMC of DTMUV-infected and non-infected control ducks are shown as lines. All viral load data were retrieved from a previous DTMUV pathogenesis study (Ninvilai et al., 2020). (D) Correlations of non-T and B lymphocyte/myeloid cell numbers with viral loads in blood and target organs. "ns" indicates not significant.

The dynamic changes of non-T and B lymphocyte numbers were analyzed in 27-week-old adult laying ducks. These non-T and B lymphocytes, characterized as CD3^-^IgY^-^, were referred to as myeloid cell subpopulation (Thontiravong et al., 2022). The myeloid subpopulation includes cells from both the monocytic series, including monocytes and dendritic cells, and the granulocytic series (Hao et al., 2020). Notably, the number of myeloid cells in DTMUV-inoculated adult laying ducks remained consistently stable, showing no significant difference compared to the non-infected control group throughout the observation period (Fig. 3A-C). Furthermore, there was no significant correlation between the numbers of myeloid cells and viral loads in the blood, spleen, and brain of 27-week-old adult laying ducks (Fig. 3D). This finding contrasted with our previous study in 4-week-old young ducks, where DTMUV infection markedly decreased myeloid cell numbers early on, but later resulted in a significant increase by 9 dpi, potentially correlating with an increased risk of severe clinical symptoms in infected younger ducks (Thontiravong et al., 2022). It is well known that the myeloid subpopulation plays a critical role in producing proinflammatory factors that contribute to the progression of illness following microbial infection (Broxmeyer and Williams, 1988). Therefore, the mild clinical disease observed in 27-week-old adult laying ducks may be partly attributed to a well-balanced myeloid cell response.

### Dynamics of neutralizing antibodies and their correlations with DTMUV loads in 27-week-old adult laying ducks

**Fig. 4:**
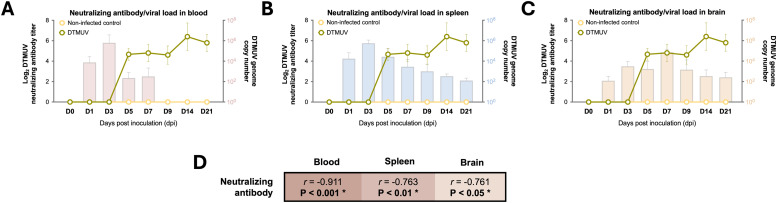
Neutralizing antibody responses of 27-week-old adult laying ducks infected with duck Tembusu virus (DTMUV). (A-C) Kinetics of DTMUV-specific neutralizing antibody titers of DTMUV-infected and non-infected control ducks at the indicated time points. The relationships between viral loads in blood (A) and target organs (B-C), alongside neutralizing antibody responses, are shown throughout the course of DTMUV infection. DTMUV genome copy numbers in blood and target organs are displayed as bars, while the levels of DTMUV-specific neutralizing antibodies in the serum of DTMUV-infected ducks are shown as lines. All viral load data were retrieved from a previous DTMUV pathogenesis study (Ninvilai et al., 2020). (D) Correlations of neutralizing antibody titers with viral loads in blood and target organs. Asterisks (*) indicate significant differences (P < 0.05, P < 0.01, and P < 0.001).

To investigate the dynamics of DTMUV-specific humoral immune responses in inoculated 27-week-old adult laying ducks, the titers of DTMUV-specific neutralizing antibodies were evaluated. The neutralizing antibodies were detected as early as 5 dpi, peaked at 14 dpi, and maintained until the end of the observation period (21 dpi) (Fig. 4A-C). The peak neutralizing antibody titer among 27-week-old adult ducks was 2^6.5±1.17^ (Fig. 4A-C), which was higher than that observed in 4-week-old young ducks (2^5.4±0.41^) (Thontiravong et al., 2022). To evaluate the association of DTMUV-specific neutralizing antibody titer with DTMUV control, the correlations between the levels of neutralizing antibodies and DTMUV loads in blood and target tissues (spleen and brain) were analyzed. In 27-week-old ducks, significantly negative correlations were observed between neutralizing antibody titers and viral loads in blood (*r* = -0.911, P < 0.001), spleen (*r* = -0.763, P < 0.01), and brain (*r* = -0.761, P < 0.05) from 5 to 21 dpi, indicating that increased neutralizing antibody titers significantly coincided with reduced viremia and viral loads in these target tissues (Fig. 4D). Contrary to previous results in 4-week-old ducks, a negative association was observed only between neutralizing antibody titers and viral loads in the blood and spleen, with no significant correlation found in the brain (Thontiravong et al., 2022). Altogether, the induction of a high neutralizing antibody titer observed in 27-week-old adult laying ducks may partly contribute to a reduced DTMUV loads and their resistance against DTMUV-induced neurological diseases.

## Discussion

**Fig. 5:**
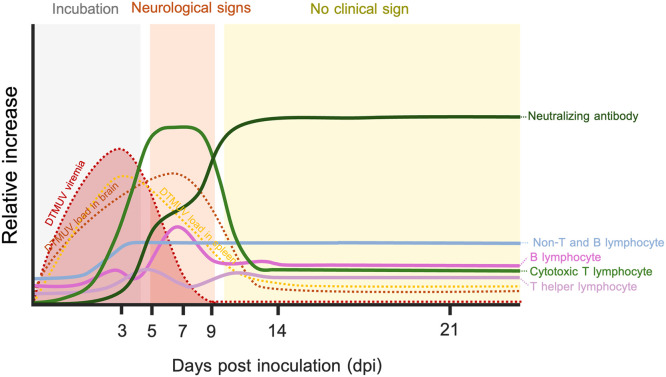
Schematic diagram of immune response dynamics in 27-week-old adult laying ducks infected with duck Tembusu virus (DTMUV). The numbers of non-T and B lymphocytes/myeloid cells in DTMUV-inoculated adult laying ducks remained consistently stable, which was likely associated with mild systemic inflammation. An increase in cytotoxic T lymphocyte (CTL, CD3^+^CD8^+^) numbers was detected between 5 and 7 dpi, returning to baseline level by 9 dpi. The numbers of both T helper lymphocytes (Th, CD3^+^CD4^+^) and B lymphocytes (CD3^-^IgY^+^) appeared to remain stable throughout the observation period. A robust neutralizing antibody response was observed as early as 5 dpi, peaked at 14 dpi, and remained at a high level until 21 dpi. Notably, the increase in CTL numbers and neutralizing antibody levels correlated with reduced viral loads in the blood, spleen, and brain. This figure includes data from this study and from a previous DTMUV pathogenesis study (Ninvilai et al., 2020).

Age is a significant factor influencing the susceptibility of ducks to DTMUV infection, leading to differences in disease outcomes. Consistent with previous observations (Sun et al., 2014; Li et al., 2015), we showed that younger ducks exhibit more severe disease and higher mortality rates than older ducks following DTMUV infection (Ninvilai et al., 2020). These age-related differences in disease severity may be linked to immune mechanisms, though the specific immunological factors remain unclear. In the present study, we investigated the dynamics of immune responses following DTMUV infection in 27-week-old adult laying ducks and compared them to our previous report on 4 weeks old young ducks (Thontiravong et al., 2022). Our results revealed that the number of myeloid cells in 27-week-old adult laying ducks infected with DTMUV remained consistently stable throughout the observation period, in contrast to findings in 4-week-old younger ducks, where myeloid cell responses were implicated in disease progression. For lymphocyte responses, unlike in 4-week-old younger ducks, only CTL responses in 27-week-old older ducks showed a significant negative correlation with the reduction of viremia and viral loads in target organs, indicating their role in controlling viral replication in older ducks. Additionally, 27-week-old ducks infected with DTMUV exhibited high levels of neutralizing antibodies, which were significantly correlated with reduced viral loads in blood and target organs. Overall, these findings highlight the age-related differences in immunological responses following DTMUV infection, which potentially contribute to the varying disease severity among ducks of different ages. A schematic diagram of the dynamics of immune responses in DTMUV-infected 27-week-old ducks is shown in Fig. 5. To the best of our knowledge, this study is the first study to provide a detailed profile of immune responses in 27-week-old adult laying ducks following DTMUV infection and to reveal the influence of age-related immune variations on DTMUV disease severity.

Our findings showed that robust DTMUV-specific neutralizing antibody and CTL responses were well correlated with reduced viral loads in the blood and target organs (spleen and brain) of infected 27-week-old ducks. The data suggest that, similar to other flaviviruses (Kreil et al., 1998; Slon Campos et al., 2018), the antibody-mediated neutralization of viruses likely plays an important role in clearing the virus from the blood and limiting its spread following infection. In addition to neutralizing antibodies, the CTL response appears to be a key component of the immune protections against systemic DTMUV infection in 27-week-old ducks. CTL target and eliminate virus-infected cells, thereby preventing the spread of virus to adjacent susceptible cells (Barry and Bleackley, 2002). It had been well-documented that strong flavivirus-specific CTL responses were markedly observed in convalescent peripheral blood of the non-fatal patients (Konishi et al., 1995). Flavivirus-specific T cell epitopes have been identified in viral structural and non-structural proteins, engaging CD8^+^ (CTL) subset proliferation in infected human (Dos Santos Franco et al., 2019). Previous studies showed that cross-reactive anti-flavivirus CTL responses contribute to protection against heterotypic infections in mouse models (Zellweger et al., 2015), and vaccine-induced CTL responses have shown promising efficacy in providing broad protection against flavivirus infections (Cunha-Neto et al., 2017). Altogether, our results suggest that effective adaptive immune responses, particularly neutralizing antibodies and CTL, in 27-week-old adult laying ducks may help prevent viral entry into the brain, thereby controlling viral loads in this critical target organ. However, the observed increase in CTL in infected ducks reflected only a cumulative response in the peripheral blood. To further understanding of immunological defenses against DTMUV-associated neurological diseases, additional investigation into the dynamics of DTMUV-specific CTL in the brains of 27-week-old ducks is essential.

In contrast to the finding in 4-week-old ducks (Thontiravong et al., 2022), the magnitude of Th (CD4^+^) lymphocyte responses observed in DTMUV-infected 27-week-old ducks were not associated with a decrease in viremia and viral load in target organs. However, although a study on human flaviviruses showed that the virus can infect CD4^+^ T lymphocytes and lead to reduced CD4 expression in infected patients (Xiang et al., 2009), the generation of virus-specific CD4^+^ T lymphocytes remains critical for achieving protective, long-lasting immunity against natural flavivirus infections (Aberle et al., 2018; Li et al., 2018). It has been well known that Th lymphocytes assist B cells in producing high-affinity neutralizing antibodies (Luckheeram et al., 2012; Aberle et al., 2018). Based on our knowledge, we believe that Th lymphocytes play a vital role in controlling viral replication and managing disease-related DTMUV infection. As mentioned in CTL findings, a significantly change of Th lymphocyte responses of 27-week-old ducks may occur in tissue-specific condition. Therefore, further investigation into the dynamic responses of DTMUV-specific Th lymphocytes in target organs of 27-week-old ducks is warranted.

Our earlier study revealed that 4-week-old young ducks infected with DTMUV exhibited a decline in myeloid cell (non-T and B lymphocyte) numbers and phagocytic activity in the early phase of infection, followed by upregulation at later time points (Thontiravong et al., 2022). It is well known that peripheral myeloid cells in the bloodstream play a crucial role in recognizing viral particles, virus-infected cells, and tissues damaged by viruses (Stegelmeier et al., 2019). These sentinels rapidly initiate an antiviral response, mounting an effective innate defense against viral infection (Stegelmeier et al., 2019). Our findings in young ducks suggest that early antiviral innate immune functions were inadequate, potentially allowing robust DTMUV replication during the initial phase of infection. Consistent with the observation, several evasion strategies have evolved in DTMUV to suppress innate immunity and facilitate productive infection, including inhibition of toll-like receptor (**TLR**) 3-mediated innate immune response and type I interferon (**IFN**) production (Li et al., 2020). Additionally, the markedly elevated levels of myeloid cells occurring later in the infection may trigger systemic inflammatory responses, thereby exacerbating disease severity in young ducks. Supporting this notion, in response to various microorganisms, myeloid cells release high levels of proinflammatory cytokines such as interleukin (**IL**)-1, IL-6, and tumor necrosis factor (**TNF**)-α leading to systemic inflammation and worsened clinical outcomes (Iqbal et al., 2016). Furthermore, a previous study showed that the dysregulation of myeloid cell signaling and activation pathways during persistent viremia of SARS-CoV-2 lead to excessive production of proinflammatory cytokines and inflammatory cell death, causing cytokine storm and organ injury in severe cases (Karki and Kanneganti, 2022). Contrast to the observation in 4-week-old young ducks, it is noteworthy that no fluctuation was observed in the myeloid subpopulation of DTMUV-infected 27-week-old adult laying ducks throughout the observation period. This finding implies that the innate immunity in older ducks is more proficient, promoting virus elimination in the early phase of infection. Moreover, a stable myeloid response during the late stage of DTMUV infection might help limit generalized inflammation, correlating with the mild systemic diseases observed in older ducks. Therefore, the variability in clinical manifestations among ducks of different ages following DTMUV infection is likely influenced by innate immune responses.

In conclusion, our findings highlight the impact of age on immunological responses to DTMUV infection, likely influencing the differences in disease severity observed among ducks of different ages. This study advances our understanding of DTMUV pathogenesis and immunity in ducks, emphasizing the interplay between age-dependent susceptibility, innate immune competence, and adaptive immune responses. The presence of robust DTMUV-specific neutralizing antibody and CTL responses is crucial for preventing viral dissemination and protection against neurological complications in infected adult laying ducks. Additionally, finely tuned myeloid cell responses may play a significant role in controlling systemic clinical signs following DTMUV infection. These insights enhance our knowledge on veterinary virology and immunology, contributing to the development of targeted immunological strategies and innovative vaccines to mitigate the impact of DTMUV infections.

## Declaration of competing interest

The authors declare that they have no conflict of interest.
